# Special Issue “New Insights into Pathophysiology, Diagnosis and Treatment of Tinnitus”

**DOI:** 10.3390/brainsci12101330

**Published:** 2022-09-30

**Authors:** Pedro Cobo, María Cuesta

**Affiliations:** Institute for Physical and Information Technologies (ITEFI), Spanish National Research Council (CSIC), 28006 Madrid, Spain

We are honoured to have been involved with Brain Sciences in the production of the Special Issue “New Insights into Pathophysiology; Diagnosis and Treatment of Tinnitus” aiming to address recent advances in the field of tinnitus.

Tinnitus can be defined as a sound arising exclusively within one’s own neural auditory system, without an external or internal sound that generates it [1]. Although a variable prevalence of tinnitus is found in the literature, tinnitus affects approximately 10–15% of the adult population [2,3], having a severe impact on the daily life of about 0.5–2% of adults and producing effects that can range from annoyance, irritation, or disturbing sleep patterns to panic, stress, anxiety, or depression [3,4,5]. Despite many articles being published on its pathophysiology, diagnosis, and treatment, the precise generation, measurement, and remedy of tinnitus remains to be completely elucidated [6].

The perception of tinnitus is due to plastic attempts of the neural auditory system to compensate for a diminished sensory input [7]. Functional imaging and electrophysiological measurements suggest that tinnitus is produced by increased neural synchrony (hypersynchrony), reorganization of the tonotopic map, and increased spontaneous firing rate (hyperactivity) of the auditory system [8].

Tinnitus can be measured by a mix of subjective and objective techniques [6]. Subjective measures of tinnitus include psychoacoustic tests, rating scales and questionnaires. Different questionnaires have been provided to assess specific aspects of tinnitus. The 25-item Tinnitus Handicap Inventory (THI) [9,10] is the most widely used tinnitus questionnaire. More recently, the Tinnitus Functional Index (TFI) has been proposed as a more sensitive questionnaire to small changes in tinnitus treatments [11,12].

Objective measures of tinnitus are urgently needed to improve diagnosis. Some neural activity mapping tools, such as electroencephalograms or functional magnetic resonance imaging, which could help assess the functionality of the auditory system are available; however, they are not feasible for regular audiological clinics due to their high costs. Another tool, the auditory evoked potential, is frequently used. It consists of recording on the scalp the electrical activity elicited by acoustic stimuli delivered at the input of the external ear. The first part of the auditory-evoked potential waveform contains the auditory brainstem response (ABR). The latency and amplitude of ABR waves are defined by the discharge rates and number of synchronously firing neurons in corresponding anatomical structures along the auditory pathway. Dehmel et al. [13] proposed that tinnitus could be objectively detected by looking for changes in discharge rate and synchrony in altered ABR waveforms. A recent meta-analysis by Milloy et al. [14], however, concluded that although some studies showed slight changes in amplitude and/or latency for high-intensity stimulation levels, these differences were not significant enough for diagnosis purposes. These results have disregarded the use of ABR as an objective tool to quantify tinnitus. However, after reviewing recent developments concerning the basis of tinnitus, Knipper et al. [15] suggested that improved ABR wave analysis should help in the future for the objective diagnosis of tinnitus.

Although tinnitus is commonly an auditory symptom, it can occur with other psychological, psychosomatic, and/or psychiatric comorbidities. Usually, the higher the level of distress, the more likely comorbid disorders are present [16]. The finding of this multifactorial relationship between a subset of personality, psychosomatic, and/or psychiatric factors and tinnitus distress is a matter of intensive current research [17,18].

Tinnitus treatments could be firstly classified in psychological and sound therapies, based on the cognitive and the neurophysiological models of tinnitus, respectively [16]. Sound therapies are currently applied jointly with counselling [19]. Many sound therapies have been proposed for tinnitus treatment [20]. According to Eggermont [20], any sound that does not annoy, create discomfort, or damage hearing is better than silence for tinnitus relief. However, sound stimuli customized to the tinnitus pitch or to the hearing loss of the subject have more therapeutic potential.

In particular, the articles collected in this Special Issue address new approaches to the pathophysiology of tinnitus [21], so as to understand where and how tinnitus is generated; to the diagnosis of tinnitus, with both objective [22,23] and subjective [24,25] techniques; and to the performance innovative treatments [26] that could contribute to alleviate the most severe effects in patients

A comprehensive understanding of the pathophysiology of tinnitus is still challenging for clinical practice. Recent theories on the tinnitus mechanism are mainly focused on the anomalous activity of the central auditory system. Al-Rawashdeh et al. proposed an outstanding mechanism of tinnitus, based on the quantum tunnelling of ion model [21]. The energy barrier of the gate is decreased by the risk factors of tinnitus promoting neuron demyelination and enhancing the quantum tunnelling of calcium, potassium, and sodium ions through the closed voltage-gated channels. Their mathematical model addresses the ability of these ions to induce the depolarization of both the inner hair cells membrane and the auditory pathway neurons. Depending on the depolarization degree, the membrane can be hyper- or hypo-excited. The inhibitory effect of depolarization (suppression of the spontaneous activity of the cochlea) was predicted. This is an important result as inhibitory effects are related to HL-induced tinnitus. The model also described quantum tunnelling signals, or quantum synapse, between the demyelinated neurons of the auditory pathway. These quantum synapses induce hyper-excitability in auditory pathway neurons and impair the signals transmitted to the central auditory system. Accordingly, this aberrant coding of these sound signals is perceived as tinnitus.

As mentioned above, reliable methods for the objective diagnosis of tinnitus are lacking at present. Electrophysiological measures, such as auditory pathway recorded potentials (EEG, ABR, MRI) are rarely used in clinical assessment, as they do not provide objective cues with necessary sensitivity and specificity to identify tinnitus. This is the subject of a first paper in this issue. Turner et al. present an observational study with 43 human subjects, 21 with and 22 without tinnitus [22]. A subgroup of 19 young adults with normal audiograms from 125 Hz to 8 kHz is also used. The ABR was measured using clicks at 1 kHz, 4 kHz, and 8 kHz, and tone bursts at 30, 50, and 70 dB nHL. Compared to control subjects, tinnitus subjects did not show reduced ABR wave I amplitude or slope in either the entire group of 21 tinnitus subjects. Turner et al. concluded that, in concordance with the results of Milloy et al. [14], the clinical use of ABR limit in diagnosing tinnitus in humans is restricted due to technical limitations.

A second paper in this subject by Fan and Li reviewed several electrophysiological approaches to detect the presence of tinnitus by analysing the change of neural activity throughout the auditory pathway [23]. They also report that factors such that the co-occurrence of hyperacusis and hidden hearing loss could have hindered the use of both wave I amplitude reduction and wave V/I amplitude ratio increase in the ABR as objective markers of tinnitus. Some other factors, such as the tinnitus aetiology, the demographic characteristics of the population, or the experimental setup (electrodes position, etc.), can disturb also the measured ABR. Therefore, this interesting review outlined the need to find suitable methods for different subtypes of tinnitus under specific stimulation modes.

Fackrell et al. provide a paper on the topic of tinnitus assessment using the Tinnitus Functional Index (TFI) as a useful questionnaire to assess changes over time related to tinnitus treatment [24]. They describe a robust score, the Minimal Important Change (MIC), in a longitudinal validation study with 255 patients affected by tinnitus. By integrating both anchor-based and distribution-based techniques, they identified an MIC score of -14 points for the TFI. Accordingly, they recommend to achieve a minimum TFI reduction of 14 points in both longitudinal studies and clinical practice.

In their interesting paper, Brueggemann et al. examine the psychosocial aspects that could help to predict tinnitus-related distress in a large dataset of chronic tinnitus patients [25]. Factor analysis is applied to group significant items related to tinnitus-related distress. This analysis revealed stress, pain experience, fatigue, autonomy, and low educational level as the five factors related to tinnitus distress. Depressive exhaustion with somatic expressions such as somatization, general psychological stress, sleep and concentration problems, and reduced activity, in addition to higher age, seemed to be the most relevant factors.

Its heterogeneity makes tinnitus difficult to treat. Tinnitus Retraining Therapy (TRT), widely used in clinical practice, combines counselling with sound therapy for tinnitus treatment [19]. TRT uses currently broadband noise as the sound stimulus. Cuesta et al. demonstrated that compensating the hearing loss curves of tinnitus subjects in the broadband sound stimuli (Enriched Acoustic Environment-EAE) improves the efficacy of conventional TRT [26]. This EAE therapy provides a greater distress reduction, which was statistically significant and clinically relevant, in a shorter period of time. Tinnitus subjects were subjected to a combination of an initial counselling session and four-month sound therapy with broadband sound stimuli coloured by their audiometry. After 4 months, 96% of these subjects achieved an average decrease of 23 point in their THI score. Furthermore, this THI reduction was dependent on the initial score, being greater in patients with higher initial tinnitus severity.

In conclusion, this Special Issue provides outstanding advances in the understanding of the pathophysiology of tinnitus, the design of reliable methods for subjective and objective tinnitus assessment and more effective treatments to alleviate tinnitus distress. We would like to thank authors for their relevant contributions and hope this reading inspires future advances in tinnitus management.

## Data Availability

Not applicable.
